# Supplementary material for: Realistic coupling enables flexible macroscopic traveling waves in the mouse cortex
Source: eLife. 2026 Sep 4;14:RP108208. doi: 10.7554/eLife.108208 (PMC13544912; doi:10.7554/eLife.108208)
Supplement: Peer review history

# Editor report: eLife Assessment

**DOI:** 10.7554/eLife.108208.3.sa0

**Authors:** Aaron Kuan

**Affiliations:** Yale School of Medicine United States

**Keywords:** Convincing, Important

This **important** work presents a novel computational framework for modeling macroscopic traveling waves in the mouse cortex by integrating open-source connectomic and transcriptomic data into a spiking network model. This approach allows the computational model to assign excitatory/inhibitory connections based on neurotransmitter profiles and extends simulations to the 3D domain. The authors present results that demonstrate how spatiotemporal dynamics such as slow oscillations (0.5-4 Hz) emerge and self-organize at the whole-brain scale. This study provides **convincing** initial insights into the structural basis of traveling waves at the whole-brain scale, and allows future links between connectome-driven spiking neural networks and whole-hemisphere imaging in the mouse.

# Referee report: Reviewer #1 (Public review)

**DOI:** 10.7554/eLife.108208.3.sa1

I thank the authors for their thoughtful and thorough responses, which address my concerns. Their two methodological changes: (1) the switch to Poisson stimulation and (2) the new LFP estimation pipeline, together with the expanded parameter-grid sweep and Kuramoto synchrony analysis, substantially strengthen the manuscript. The Poisson spike train better approximates the stochastic subcortical drive cortex receives in vivo and removes the artificiality of the original protocol (Point 1.2). The LFP pipeline directly resolves my concern about the disconnect between simulated voltages and experimental signals; showing that the macroscopic wave structure persists in the LFP-like proxy clarifies the framework's practical relevance (Point 1.6). The expanded per-band sweep addresses my worry that the Allen-connectivity advantage was confined to a narrow regime, and acknowledging the small delta-band difference is a more convincing presentation (Point 1.5). The Kuramoto analysis connects dynamics across scales and gives a clear, quantitative account of the non-monotonic coupling dependence (Points 1.4, 1.7). Finally, I appreciate that the remaining connectivity-realism issues (Points 1.3, 1.8) are now stated explicitly as limitations with concrete future directions. I agree that incorporating them is beyond the scope of the present study, and their upfront acknowledgement is appropriate.

# Referee report: Reviewer #2 (Public review)

**DOI:** 10.7554/eLife.108208.3.sa2

Summary:

This work presents a spiking network model of traveling waves at the whole-brain scale in mouse neocortex. The authors use data from the Allen Institute to re-construct connectivity between different neocortical sites. They then quantify macroscopic traveling waves following stimulation of all layer 4 neurons in neocortex.

Strengths:

Overall, the results are interesting and shed new light on the dynamic organization of activity across neocortex of the mouse. The paper uses realistic neuron models specifically fit to intracellular recordings, demonstrating that traveling waves occur in the mouse neocortex with both realistic connectivity and realistic single-neuron dynamics. The paper is also well-written in general. For these reasons, the authors have generally achieved their aims in this work.

Weaknesses:

(1) Description of Algorithm 1: While the Methods section clearly explains the density parameter \rho, the statement on line 358 concerning the "ideal" average number of connections is a little unclear. The authors should explicitly clarify that \rho is a free parameter that can be adjusted to balance computational feasibility (for a given set of computational resources) and biological fidelity.

(2) Lines 102-103: The \rho parameter used here results in approximately 300 connections per neuron on average. The authors should state clearly that the number of connections per cell is the key determinant of computational feasibility (cf. Morrison et al., Neural Computation, 2005). The authors should also review neuronal density and synaptic connectivity in mouse neocortex and clearly reference density and connectivity in their model to the biological scales found in the mouse.

(3) Line 131: From the plots in Figure 2, it is not clear that the stimulus response is necessarily a rhythmic oscillation, in the sense of a single narrowband frequency.

(4) Line 217: Can the authors clarify how these findings relate to the results from Mohajerani et al. (Nature Neuroscience, 2013), or differ from them?

(5) Line 230: Because higher temporal frequency activity also tends to be more spatially localized, a correlation between PGD and temporal frequency could be an inherent consequence of this relationship, rather than a meaningful result.

(6) Line 247-248: It is not clear that the algorithm for generating connections between neurons presented here really relates to those for community detections. For example, in the case of the Allen Institute data, the communities are essentially in the data already.

(7) Line 284-285: The relationship between conduction delay is more direct than this sentence suggests. Conduction delay is fundamentally determined by the time required for action potentials to propagate along axons, making it intrinsically linked to anatomical distance.

(8) Line 287-288: The authors suggest at this point that they do not have enough information to estimate time delays due to axonal conduction along white matter fibers. However, experimental data from white matter connections typically includes information about fiber length, which does enable estimating conduction delays. These estimations have been previously implemented for Allen Institute connectome data in the mouse (Choi and Mihalas, PLoS Comput Biology, 2019) and human connectome data (Budzinski et al., Physical Review Research, 2023).

(9) Lines 294-295: Several methods do exist for detecting and characterizing wave dynamics in three-dimensional data (Budzinski et al., Physical Review Research, 2023).

Comments on revised version.

In this response and revised manuscript, the authors have addressed all points raised in the first round of review. In response to Point 2.7, however, is it not the case that the Allen dataset has the axonal lengths?

# Author comment: Author response

**DOI:** 10.7554/eLife.108208.3.sa3

**Authors:** Guanhua Sun, James Hazelden, Ruby Kim, Daniel B Forger

**Affiliations:** University of Michigan Ann Arbor United States; University of Michigan Ann Arbor United States; University of Michigan Ann Arbor United States; University of Michigan Ann Arbor United States

The following is the authors’ response to the original reviews.

**Public Reviews:**

**Reviewer #1 (Public review):**

(1.1) The manuscript “Realistic coupling enables flexible macroscopic traveling waves in the mouse cortex” by Sun, Forger, and colleagues presents a novel computational framework for studying macroscopic traveling waves in the mouse cortex by integrating realistic brain connectivity data with large-scale neural simulations.

The key contributions include: (1) developing an algorithm that combines spatial transcriptomic data (providing detailed neuron positions and molecular properties) with voxelized connectivity data from the Allen Brain Atlas to construct neuron-to-neuron connections across 300,000 cortical neurons; (2) building a GPU-accelerated simulation platform capable of modeling this large-scale network with both excitatory and inhibitory HodgkinHuxley neurons; (3) extending phase-based analysis methods from 2D to 3D to quantify traveling wave activity in the realistic brain geometry; and (4) demonstrating that realistic Allen connectivity generates significantly higher levels of macroscopic traveling waves compared to simplified local or uniform connectivity patterns.

The study reveals that wave activity depends non-monotonically on coupling strength and that slow oscillations (0.5-4 Hz) are particularly conducive to large-scale wave propagation, providing new insights into how anatomical connectivity enables flexible spatiotemporal dynamics across the cortex.

The authors leverage two existing dense datasets of spatial transcriptomic data and connection strength between pairwise voxels in the mouse cortex in a novel way, allowing for the computational model to capture molecular and functional properties of neurons as determined by their neurotransmitter profiles, rather than making arbitrary assignments of excitatory/inhibitory roles. Additionally, the author’s expansion of 2D phase dynamics to 3D phase gradient analysis methods is important and can be widely applied to calcium imaging, LFP recordings, and likely other electrophysiological recordings.

Thank you for the accurate summary of our manuscript and list of strengths.

(1.2) The model’s Allen connectivity approach overlooks critical aspects of real cortical dynamics. Most importantly, it excludes subcortical structures, especially the thalamus, which drives cortical traveling waves through thalamocortical interactions. The authors’ method of electrically stimulating all layer 4 neurons simultaneously to initiate waves is artificially crude and bears little resemblance to natural wave generation mechanisms.

We agree that excluding subcortical structures, especially the thalamus, is an important limitation of the current model. Because adding these structures would substantially expand the scope and complexity of the model, we now state this limitation more explicitly in the Discussion and leave it as a future extension:

“For simulations, we choose to randomly stimulate the total population of layer 4 neurons as a way to mimic subcortical input and generate traveling waves, which can be unrealistic. Subcortical structures, such as the thalamus, are vital to cortical dynamics like slow-wave activity [1] and are known to regulate traveling waves [2]. Therefore, a direct and important future improvement would be adding subcortical structures to the model.”

We also agree that the original constant-current stimulation was too artificial. We therefore replaced it with a 10Hz Poisson spike train delivered to layer-4 excitatory neurons across the isocortex, which more closely mimics the stochastic input that cortex receives from subcortical regions such as the thalamus. The revised stimulation protocol is described in the Results:

“Therefore, to mimic the stochastic drive that cortex receives from subcortical regions like thalamus, we deliver a 10Hz Poisson spike train to layer-4 excitatory neurons (Figure 2a), since layer 4 is the canonical thalamocortical input layer [3]; each Poisson event applies a fixed voltage bump *V*_stim_, and no other external input is applied.”

as well as in the Methods 4.5 Simulation.

The revised stimulation protocol allowed us to rerun the parameter sweep under stochastic drive. A direct comparison of alternative wave-generating mechanisms remains an important direction for future work.

(1.3) The model handles voxel-to-voxel connections crudely when neurons have mixed excitatory/inhibitory properties and varying synaptic strengths. Real connectivity differs dramatically between neuron types (pyramidal cells vs. interneurons, across cortical layers), but the model only distinguishes excitatory and inhibitory neurons. Additionally, uniform synaptic weights ignore natural variations in connection strength based on neuron type, distance, and functional role. Integrating the updated thalamocortical dataset mentioned by the authors, even at regional resolution, would substantially improve the model.

We thank the reviewer for raising these important points regarding cell-type-specific connectivity and heterogeneity of synaptic weights. We agree that cell-type-specific connectivity and heterogeneous synaptic weights are important limitations. Because the current voxelized projectome is not cell-type specific, we now state these limitations explicitly and outline how future versions of the model could incorporate improved density and synaptic weight assumptions in the Discussion (Construction of the Allen connectivity). Specifically, we now write:

“First, the voxelized projection data is not cell-type specific. In our model, we distinguish only two neuronal populations: glutamatergic (excitatory) and GABAergic (inhibitory), based on the Zhuang-ABCA-1 transcriptomic dataset. However, real cortical connectivity differs dramatically between more refined cell types: pyramidal neurons and interneurons have distinct connection targets, and connectivity is strongly layer-specific. At the same time, we use a single global density parameter *ρ* to set the average number of connections per neuron across the entire cortex. A region-specific *ρ* could better capture the known variation in local synaptic density across cortical areas, for example the higher synaptic density of primary sensory regions relative to higher-order association areas. Future versions of the model could allow a region- and cell-type-specific *ρ* derived from region-level synapse-density atlases together with cell-type-resolved connectivity [4], which would simultaneously address the limitations noted above.

Also, our model uses uniform synaptic weights within each synapse type: every AMPA synapse has conductance *g*_AMPA_ and every GABA synapse has conductance *g*_GABA_. In reality, synaptic strength varies with presynaptic and postsynaptic cell type, anatomical distance, and the distribution of synaptic weights is typically heavy-tailed (e.g. log-normal [5]). Extending our algorithm to sample synaptic weights from realistic distributions would be a natural next step and is likely necessary for quantitative comparisons with electrophysiological recordings.”

These additions clarify which aspects of the current model are constrained by the available voxelized projectome and which extensions would require cell-type-resolved connectivity data, region-specific density information, or more detailed synaptic-weight estimates.

(1.4) While the authors bridge microscopic (single neuron) and mesoscopic (regional connectivity) data to study macroscopic (whole-cortex) waves, they don’t integrate the distinct mechanisms operating at each scale. The framework demonstrates that realistic connectivity enables macroscopic waves but fails to connect how wave dynamics emerge and interact across spatial scales systematically.

We thank the reviewer for this insightful comment. In the revision, we added the Kuramoto synchrony analysis as a first step toward connecting scales: changes in the microscopic coupling parameter alter network synchrony, and this synchrony measure is closely associated with the macroscopic PGD observable (new Fig. 5). We agree that a full separation of layer-specific microcircuit mechanisms, region-specific connectivity motifs, and whole-cortex wave propagation remains beyond the scope of the present study. The revised text now frames the synchrony-to-wave relationship as one concrete cross-scale link that can be investigated further with this framework.

(1.5) Claims that Allen connectivity produces higher phase gradient directionality (PGD) than local connectivity appear limited to delta oscillations at very specific coupling strengths and applied currents. Few parameter combinations show significantly higher PGD for Allen connectivity, and these are generally low PGD values overall.

We agree that the original comparison did not sufficiently establish whether the Allen-connectivity advantage held beyond a small number of parameter choices.

In the revised manuscript, we expanded the analysis to a 10 × 12 grid of excitatory coupling strengths and Poisson stimulus magnitudes for Allen, local, and uniform connectivity. Across this full (*g*_AMPA_,*V*_stim_) grid, Allen connectivity shows higher per-band maximum PGD than local or uniform connectivity, especially in the theta, alpha, and beta bands (Fig. 5b). The delta-band difference is small, consistent with the reviewer’s observation that the original delta-band result did not clearly separate Allen from local connectivity.

In the revised manuscript, Fig. 5c and Fig. 5d show how PGD varies with stimulus magnitude and coupling strength individually. The full per-band PGD heatmaps for all three connectivities, together with the per-band Allen-minus-opponent gap bars, are provided in Figure 4-figure supplement 6. These additions show that the Allen-connectivity trend is not limited to a single representative operating point.

(1.6) Broadly, it’s unclear how this computational framework can study memory, learning, sleep, sensory processing, or disease states, given the disconnect between simulated intracellular voltages and the local field potentials or other electrophysiological measurements typically used to study cortical traveling waves. While computationally impressive, the practical research applications remain vague.

We thank the reviewer for this important point. To bridge the gap between our simulations and experimentally measured data, such as local field potentials (LFP), we used a post-hoc LFP estimation pipeline and added a dedicated Methods subsection describing it (LFP estimation from intracellular voltage). The key idea is that LFP primarily reflects the net transmembrane synaptic current in a local population, which we can reconstruct directly from the voltage traces and the connectivity used in the simulation. Please see Methods section LFP estimation from intracellular voltage.

This pipeline allows us to test whether the macroscopic traveling-wave structure identified in the voltage traces is also present in an LFP-like signal. In the revised manuscript, we added a new Results section and show, in Fig. 6, side-by-side voltage- and LFP-based snapshots, the voltage-LFP PGD scatter (Pearson *r* = 0.78-0.89 per band), and per-band PGD comparisons across Allen, local, and uniform connectivity on the LFP signal. These results indicate that our conclusions are not restricted to intracellular voltage and provide a closer bridge to LFP-based experimental measurements.

(1.7) The paper needs a clearer explanation for why medium coupling (100%) eliminates waves in Allen connectivity (Figure 6) while stronger coupling (150%) restores them.

Thank you for requesting this clarification. The revised analysis suggests that the non-monotonic relationship between coupling strength and wave activity reflects an interaction between network synchrony and spatial organization. At weak coupling, the network has enough coordination to support propagating waves. At medium coupling, increased synaptic drive pushes the network into an asynchronous irregular state that disrupts coherent wave fronts. At strong coupling, rhythmic synchronization is re-established and again supports wave propagation.

To substantiate this explanation quantitatively, we measured the Kuramoto order parameter R(t)=|〈e^iϕ(x,t)^〉_x_| from the generalized phase ϕ(x, t) of the band passed voltage field (see updated Quantitative measurement of neuronal activity in Methods) and reduced it to the maximum over each 1-s recording window. We then swept the same ten *g*_AMPA_ values (0.005-0.050 nS) and twelve stimulus magnitudes used for the PGD sweeps, for Allen, local and uniform connectivity, in all five frequency bands. The new analysis is presented in Fig. 5: panel (e) shows synchrony versus *g*_AMPA_ for the three connectivities, panel (d) shows the matching PGD curve, and panel (f) shows the synchrony-PGD scatter with Pearson r per connectivity.

The synchrony curve captures the main peak-dip-recovery structure of the PGD curve. For Allen connectivity in the alpha band, mean synchrony peaks at weak coupling, collapses to a 75 % suppression in the medium-coupling window *g*_AMPA_ = 0.020-0.035 nS, and recovers near unity at *g*_AMPA_ ≥ 0.040 nS. Uniform connectivity follows the same U-shape with a slightly earlier dip. Per-band versions of the PGD- and synchrony-vs-coupling curves are provided in Figure 5-figure supplement 1, showing that the peak-dip-recovery profile holds across all five canonical bands for Allen and uniform connectivity. Across the 120- point (*g*_AMPA_, *V*_stim_) grid, synchrony and PGD are positively correlated in every band and every connectivity (Pearson *0*r ranging from ≈ 0.30 to ≈ 0.92 across band-connectivity combinations; per-band scatters in Figure 5-figure supplement 2). Local connectivity follows a different trajectory: its synchrony is moderate at weak coupling and decays monotonically with gAMPA without recovering at strong coupling. This is consistent with local connectivity not supporting large-scale propagating waves at strong coupling, so the peak-dip-recovery interpretation applies mainly to Allen and uniform connectivity. We have added this synchrony analysis to the revised manuscript:

“To diagnose the mechanism behind this profile, we measured the Kuramoto order parameter R(t) = |⟨e^iϕ(x,t)^⟩_x_| from the generalized phase field ϕ(x, t) of each frequency band and recorded its maximum over each simulation window (Quantitative measurement of neuronal activity). The resulting synchrony curve (Figure 5e) resembles the trend of the maximum PGD well (Figure 5d). For Allen connectivity in the alpha band, mean synchrony peaks at weak coupling, collapses in the medium-coupling window, and recovers at strong coupling. Across the full 120-point (*g*_AMPA_, *V*_stim_) grid, synchrony and PGD are positively correlated in every band and every connectivity (Pearson *r* = 0.30- 0.92, all *p* < 10−3; Figure 5f, with per-band scatters in figure Supplement 2). Therefore, the PGD trough at medium coupling may be a synchrony trough: increased synaptic drive pushes the network into an asynchronous irregular state, while strong coupling re-establishes rhythmic synchronization that supports wave propagation. This synchrony-to-wave bottleneck seems more significant to the networks with long-range connectivity (Allen, uniform), partly because long-range connections can augment synchrony in the coupled neuronal network [6]. We also note that although uniform connectivity is able to achieve almost an identical level of synchrony to that of Allen connectivity, the PGD remains much lower due to the loss of spatial organization within.”

(1.8) Does using a single connectivity parameter (ρ = 300) across all regions miss important regional differences in cortical connectivity density?

We thank the reviewer for raising this important point. We agree that a single global density parameter misses region-to-region variation in local synaptic density, and we have extended the Discussion (Construction of the Allen connectivity) to state this limitation alongside the cell-type-specific connectivity and synaptic-weight limitations discussed in response to Point 1.3. Specifically, we now write:

“At the same time, we use a single global density parameter ρ to set the average number of connections per neuron across the entire cortex. A region-specific ρ could better capture the known variation in local synaptic density across cortical areas, for example the higher synaptic density of primary sensory regions relative to higher-order association areas. Future versions of the model could allow a region- and cell-type-specific ρ derived from region-level synapse density atlases together with cell-type-resolved connectivity [4], which would simultaneously address the limitations noted above.”

This paragraph is placed directly after the cell-type and weight-heterogeneity limitations added in response to Point 1.3.

**Reviewer #2 (Public review):**

(2.1) This work presents a spiking network model of traveling waves at the whole-brain scale in the mouse neocortex. The authors use data from the Allen Institute to reconstruct connectivity between different neocortical sites. They then quantify macroscopic traveling waves following stimulation of all layer 4 neurons in the neocortex.

Overall, the results are interesting and shed new light on the dynamic organization of activity across the neocortex of the mouse. The paper uses realistic neuron models specifically fit to intracellular recordings, demonstrating that traveling waves occur in the mouse neocortex with both realistic connectivity and realistic single-neuron dynamics. The paper is also well-written in general. For these reasons, the authors have generally achieved their aims in this work.

We thank Reviewer 2 for the positive assessment and accurate summary of our work.

(2.2) Description of Algorithm 1: While the Methods section clearly explains the density parameter *ρ*, the statement on line 358 concerning the “ideal” average number of connections is a little unclear. The authors should explicitly clarify that *ρ* is a free parameter that can be adjusted to balance computational feasibility (for a given set of computational resources) and biological fidelity. The *ρ* parameter used here results in approximately 300 connections per neuron on average. The authors should state clearly that the number of connections per cell is the key determinant of computational feasibility (cf. Morrison et al., Neural Computation, 2005). The authors should also review neuronal density and synaptic connectivity in the mouse neocortex and clearly reference density and connectivity in their model to the biological scales found in the mouse.

We thank the reviewer for raising this important point about our connectivity algorithm and simulation. We have clarified in the revised Methods (Use of the voxelized connectivity data, Methods 4.2) that *ρ* is a free parameter that controls the trade-off between computational feasibility and biological fidelity. Specifically, we now write:

“The density parameter *ρ* is a free parameter that controls the trade-off between computational feasibility and biological fidelity: higher values of *ρ* yield more connections per neuron and thus higher biological realism, at the cost of greater memory and runtime [7].”

A careful accounting of the biological scales involved (synapse density per neuron, total cortical population) and incorporating region or cell-type-specific connection density is left as a future direction. We have noted this in the Discussion subsection:

“First, the voxelized projection data is not cell-type specific. In our model, we distinguish only two neuronal populations: glutamatergic (excitatory) and GABAergic (inhibitory), based on the Zhuang-ABCA-1 transcriptomic dataset. However, real cortical connectivity differs dramatically between more refined cell types: pyramidal neurons and interneurons have distinct connection targets, and connectivity is strongly layer-specific. At the same time, we use a single global density parameter *ρ* to set the average number of connections per neuron across the entire cortex. A region-specific *ρ* could better capture the known variation in local synaptic density across cortical areas, for example the higher synaptic density of primary sensory regions relative to higher-order association areas. Future versions of the model could allow a region- and cell-type-specific *ρ* derived from region-level synapse-density atlases together with cell-type-resolved connectivity [4], which would simultaneously address the limitations noted above.”

(2.3) Line 131: From the plots in Figure 2, it is not clear that the stimulus response is necessarily a rhythmic oscillation, in the sense of a single narrowband frequency.

The reviewer is correct, and we are grateful for the prompt to be more precise. The Results phrasing around Fig. 2 has been softened to avoid any implication of narrowband rhythmicity, and now reads:

“Under this protocol, we immediately observe macroscopic traveling waves emerge across the cortex (Figure 2 and Videos). The global mean voltage and the region-sorted raster (Figure 2c, d) reveal oscillatory activity that is well synchronized across regions, while the local-mean intracellular voltage maps over a representative 50ms window (Figure 2b) reveal a coherent wavefront sweeping along the anterior-posterior axis, consistent with previously reported cortex-wide waves [8, 9, 10]. The corresponding single-neuron-resolution view of the same simulation, with no spatial averaging, is shown in figure Supplement 1.”

Moreover, we have revised the Introduction to describe [11] as demonstrating traveling waves in broadband (5-40Hz) activity, making clear that traveling waves can occur without requiring narrowband oscillations (see also our response to your related point below). In the revised manuscript, we also separate the broadband activity into canonical frequency bands and analyze the wave activity in each band independently.

(2.4) Line 217: The authors should clarify how these findings relate to the results from Mohajerani et al. (Nature Neuroscience, 2013) or differ from them.

We thank the reviewer for this suggestion. We agree that [12] is an important experimental benchmark. A direct quantitative comparison is difficult because the original data were not aligned to the Allen Brain Atlas CCF used in our simulations. We therefore revised the Discussion to identify this comparison as a future direction, alongside the visual-cortex bidirectional-wave data of [13]:

“A more detailed quantitative comparison with experimental cortical-wave studies, such as the cortex-wide voltage-imaging data of [12] or the bidirectional visual-evoked waves reported by [13], is left as a future direction.”

(2.5) Line 230: Because higher temporal frequency activity also tends to be more spatially localized, a correlation between PGD and temporal frequency could be an inherent consequence of this relationship, rather than a meaningful result.

We thank the reviewer for raising this important point. The reviewer is correct that higher-frequency oscillations tend to be more spatially localized, which can inherently reduce PGD when measured globally. We therefore revised this analysis by separating the broadband signals into canonical frequency bands and comparing PGD within each band.

In the revised manuscript, we no longer interpret cross-band PGD differences as evidence for a frequency-to-spatial-scale relationship. Instead, we report the level of macroscopic wave activity within each canonical band. This per-band comparison is summarized in Fig. 5b, which reports the maximum PGD in each band (mean ± SEM across the entire (*g*_AMPA_,*V*_stim_) grid) for the three connectivities. Allen connectivity shows higher per-band PGD than local or uniform connectivity in the theta, alpha, and beta bands, without requiring an interpretation of PGD differences across frequency bands.

For completeness, we also computed the per-band mean(Allen) − mean(opponent) PGD gap across the full parameter grid (10 *g*_AMPA_ × 12 *V*_stim_ = 120 points per connectivity). The result is presented in Figure 4-figure supplement 6: panel (a) gives the per-band maxPGD heatmaps over the (gAMPA, Vstim) grid for each connectivity, and panel (b) gives the per-band Allen-minus-opponent mean-PGD gap. The mean gap against Local is −0.004 in delta, +0.027 in theta, +0.036 in alpha, +0.036 in beta and +0.004 in gamma, and against Uniform is +0.015, +0.044, +0.052, +0.040 and +0.011, respectively. The gap is largest in alpha and broadly concentrated in theta-alpha-beta, rather than in delta as we had originally written. We report these per-band gaps descriptively because they summarize one parameter sweep per connectivity rather than independent biological or simulation replications, and we do not interpret the differences across bands as a meaningful frequency dependence. We have added this analysis to the revised manuscript.

(2.6)Line 247-248: It is not clear that the algorithm for generating connections between neurons presented here really relates to those for community detection. For example, in the case of the Allen Institute data, the communities are essentially in the data already.

We agree with the reviewer that the relevant anatomical blocks are already present in the data. Our intent was to relate the sampling procedure to stochastic block models for network generation, not to community-detection algorithms. We have revised this passage in the Discussion subsection Construction of the Allen connectivity: ”In essence, our algorithm belongs to the family of stochastic block models [14], where the block structure is given by the voxelization of the Allen Brain Atlas and the inter-block connection probabilities are set by the voxelized projection strengths.”

(2.7) Line 284-285: The relationship between conduction delay is more direct than this sentence suggests. Conduction delay is fundamentally determined by the time required for action potentials to propagate along axons, making it intrinsically linked to anatomical distance.

Thank you for raising this important point. We agree that conduction delay is directly tied to axonal propagation time and therefore to anatomical path length. Our original wording was intended to note that the relevant path length cannot be approximated reliably by Euclidean distance in the 3-D coordinate space. We have revised this passage in the Discussion sub-section Cortical model and simulation to state that conduction delay is linked to the white-matter path length of the connection, while noting that our model lacks the actual axonal path geometry through the cortical manifold:

“However, we did not include conduction delay in our study. Conduction delay is thought to have a proportional relationship with the white matter path length of the connection between two neurons [15]. In our model, although we have the 3D positions of neurons, we do not have geometric information about the cortical manifold. Two neurons can be very close in the Cartesian coordinates measured by the Euclidean distance, but very far in terms of the length of the actual connection in the brain. Therefore, incorporating accurate conduction delay in the model is an important future direction.”

(2.8) Lines 294-295: Several methods do exist for detecting and characterizing wave dynamics in three-dimensional data (Budzinski et al., Physical Review Research, 2023).

Thank you for this reference. We have added a citation to [16] in the Discussion subsection Quantitative measurements of 3-D traveling waves, acknowledging that methods for 3-D wave analysis do exist while noting that most published algorithms are designed for 2-D data:

“There are many techniques available for identifying and measuring large-scale neuronal spatiotemporal patterns [17, 18]. While methods for detecting wave dynamics in three-dimensional data do exist [16], most published algorithms are designed to analyze 2-D data, so our simulation data, which is intrinsically 3-D, presents new challenges for measurement.”

(2.9) Line 28: It is important to note that the Davis et al. (2020) reference is not actually in the beta band, but instead in the broadband (5-40 Hz). This distinction is important because it demonstrates that waves can occur in neural data without requiring narrowband oscillations.

Thank you for this correction. We have moved the [11] citation out of the beta-band group in the Introduction and reframed it as a broadband (5-40Hz) reference, so that the sentence now reads:

“These waves are observed at different frequencies during various brain activities, ranging from slow-wave activity [8, 9], sleep spindles [19], to faster oscillations in alpha [20, 21], beta [22, 23], and gamma [21, 13] frequency bands, as well as in broadband (5-40Hz) activity [11].”

This distinction is important because it makes clear that traveling waves do not require narrowband oscillations. The revised manuscript therefore separates the broadband activity into canonical frequency bands and compares wave activity within each band.

(2.10) Line 46-49: This sentence could be clearer, for example, by specifying “certain dynamics” in more precise terms.

We apologize for the imprecision in the original manuscript. We have clarified the sentence in the Introduction to specify that the dynamics of interest are coexistence patterns of local and global activity in the network, as described in the cited reference [24]:

“It has been shown that in a coupled neuronal network, the coexistence of global wave activity with locally asynchronous states only arises when the number of oscillators is high enough, where local and global activities can coexist [24].”

(2.11) Figure 1b(i): Small typo in the label for this panel.

This has been corrected. Thank you for catching it.

(2.12) Lines 121-122: It may be important to note that spiking neural networks can also generate self-sustained activity (Vogels and Abbott, JNeurosci, 2005; Kumar et al., Neural Computation, 2008). This self-sustained activity is a form of internally generated “frozen” noise that is fundamentally different from externally imposed noise sources (such as Poisson external input) (Destexhe and Contreras, Science, 2006). Waves appear in this self-sustained activity, as well (Davis et al., Nature Communications, 2021), supporting the generality of this phenomenon.

Thank you for pointing out these important references. We have added citations to [25], [26], [27], and [24] in the Results subsection Macroscopic traveling waves emerge from random stimulation through realistic connectivity:

“Spiking networks of this scale can also generate self-sustained activity in similar regimes [25, 26], which differs fundamentally from externally imposed noise [27], and traveling waves have been reported under such conditions [24].”

In the revised manuscript, we also changed the stimulation protocol from constant current to Poisson input, which is more similar to the stochastic input that cortical neurons receive in vivo. Traveling waves observed in our model under this Poisson drive therefore complement, rather than depend on, the self-sustained-activity regime emphasized by the cited works.

(2.13) Lines 139-140: “anterior-posterior macroscopic waves in both directions” and ”in the reverse direction right after each other” could be clearer. In addition, the study from Aggarwal et al. (Nature Communications, 2022) could be relevant to note at this point.

We thank the reviewer for these wording suggestions and the relevant reference. In the revised manuscript, we reran the simulations and updated Figure 2 accordingly. The new representative simulation shown in Fig. 2 emphasizes a coherent wavefront sweeping along the anterior-to-posterior axis, and the original passages describing consecutive opposite-direction waves have been removed from the Results subsection Macroscopic traveling waves emerge from random stimulation through realistic connectivity, which now reads:

“Under this protocol, we immediately observe macroscopic traveling waves emerge across the cortex (Figure 2 and Videos). The global mean voltage and the region-sorted raster (Figure 2c, d) reveal oscillatory activity that is well synchronized across regions, while the local-mean intracellular voltage maps over a representative 50ms window (Figure 2b) reveal a coherent wavefront sweeping along the anterior-posterior axis, consistent with previously reported cortex-wide waves [8, 9, 10]. The corresponding single-neuron-resolution view of the same simulation, with no spatial averaging, is shown in figure Supplement 1.”

Bidirectional propagation can still occur in the model, but we have chosen not to present it as a focal result of this revised manuscript; consequently, the specific phrasings flagged by the reviewer no longer appear in the Results text.

We agree that [13] is an important reference, and we now discuss it in the Comparing with experimental data subsection as a potential benchmark for future quantitative comparison with our model.

(2.14) Figure 7: The caption for this figure could be clearer.

Line 281: typo ”Ermentrou”.

Line 418: typo ”excitatory”.

The typos (“Ermentrou” → “Ermentrout”, and the “excitatory” typo at line 418) have been corrected. The original Figure 7 has been removed from the revised manuscript; its content (PGD versus stimulus magnitude and coupling strength, and PGD by dominant frequency bucket) has been reorganized across the new Figs. 4-6.

References

(1) Steriade M, Mccormick DA, Sejnowski TJ. Thalamocortical Oscillations in the Sleeping and Aroused Brain. Science. 1993;262(5134):679-85.

(2) Ye Z, Bull MS, Li A, Birman D, Daigle TL, Tasic B, et al. Brain-wide topographic coordination of traveling spiral waves. BioRxiv. 2023:2023-12.

(3) Rockland KS.What do we know about laminar connectivity? Neuroimage. 2019;197:772-84.

(4) Harris JA, Mihalas S, Hirokawa KE, Whitesell JD, Choi H, Bernard A, et al. Hierarchical organization of cortical and thalamic connectivity. Nature. 2019;575(7781):195+. Available from: https://www.nature.com/articles/s41586-019-1716-z.

(5) Buzs´aki G, Mizuseki K. The log-dynamic brain: how skewed distributions affect network operations. Nature Reviews Neuroscience. 2014;15(4):264-78.

(6) Bazhenov M, Rulkov NF, Timofeev I. Effect of synaptic connectivity on long-range synchronization of fast cortical oscillations. Journal of neurophysiology. 2008;100(3):156275.

(7) Morrison A, Aertsen A, Diesmann M. Spike-timing-dependent plasticity in balanced random networks. Neural computation. 2007;19(6):1437-67.

(8) Massimini M. The Sleep Slow Oscillation as a Traveling Wave. Journal of Neuroscience. 2004;24(31):6862-70. Available from: https://doi.org/10.1523/jneurosci.1318-04.2004.

(9) Liang Y, Song C, Liu M, Gong P, Zhou C, Kno¨pfel T.Cortex-Wide Dynamics ofIntrinsic Electrical Activities: Propagating Waves and Their Interactions. The Journal of Neuroscience. 2021;41(16):3665-78. Available from: https://www.jneurosci.org/content/jneuro/41/16/3665.

(10) Aggarwal A, Luo J, Chung H, Contreras D, Kelz MB, Proekt A. Neural assemblies coordinated by cortical waves are associated with waking and hallucinatory brain states. Cell Reports. 2024;43(4):114017. Available from: https://www.sciencedirect.com/science/article/pii/S2211124724003450.

(11) Davis ZW, Muller L, Martinez-Trujillo J, Sejnowski T, Reynolds JH. Spontaneous travelling cortical waves gate perception in behaving primates. Nature. 2020;587(7834):4326. Available from: https://doi.org/10.1038/s41586-020-2802-y.

(12) Mohajerani MH, Chan AW, Mohsenvand M, LeDue J, Liu R, McVea DA, et al. Spontaneous cortical activity alternates between motifs defined by regional axonal projections. Nature Neuroscience. 2013;16(10):1426-35. Available from: https://doi.org/10.1038/nn.3499.

(13) Aggarwal A, Brennan C, Luo J, Chung H, Contreras D, Kelz MB, et al. Visual evoked feedforward–feedback traveling waves organize neural activity across the cortical hierarchy in mice. Nature Communications. 2022;13(1):4754. Available from: https://doi.org/10.1038/s41467-022-32378-x.

(14) Holland PW, Laskey KB, Leinhardt S. Stochastic blockmodels: First steps. Social Networks. 1983;5(2):109-37. Available from: https://www.sciencedirect.com/science/article/pii/0378873383900217.

(15) Lemar´echal JD, Jedynak M, Trebaul L, Boyer A, Tadel F, Bhattacharjee M, et al. A brain atlas of axonal and synaptic delays based on modelling of cortico-cortical evoked potentials. Brain. 2022;145(5):1653-67.

(16) Budzinski RC, Nguyen TT, Benigno GB, Đoàn J, Mináč J, Sejnowski TJ, Muller LE. 2023. Analytical prediction of specific spatiotemporal patterns in nonlinear oscillator networks with distance-dependent time delays. *Physical Review Research* **5**:01. 10.1103/PhysRevResearch.5.013159

(17) Townsend RG, Gong P. Detection and analysis of spatiotemporal patterns in brain activity. PLOS Computational Biology. 2018;14(12):e1006643. Available from: http://doi.org/10.1371/journal.pcbi.1006643.

(18) Gutzen R, De Bonis G, De Luca C, Pastorelli E, Capone C, Allegra Mascaro AL, et al. A modular and adaptable analysis pipeline to compare slow cerebral rhythms across heterogeneous datasets. Cell Reports Methods. 2024;4(1). Available from: http://doi.org/10.1016/j.crmeth.2023.100681.

(19) Muller L, Piantoni G, Koller D, Cash SS, Halgren E, Sejnowski TJ. Rotating waves during human sleep spindles organize global patterns of activity that repeat precisely through the night. Elife. 2016;5. Available from: https://www.ncbi.nlm.nih.gov/pubmed/27855061.

(20) Zhang H, Watrous AJ, Patel A, Jacobs J. Theta and alpha oscillations are traveling waves in the human neocortex. Neuron. 2018;98(6):1269-81. e4.

(21) van Kerkoerle T, Self MW, Dagnino B, Gariel-Mathis MA, Poort J, van der Togt C, et al. Alpha and gamma oscillations characterize feedback and feedforward processing in monkey visual cortex. Proc Natl Acad Sci U S A. 2014;111(40):14332-41. Available from: https://www.ncbi.nlm.nih.gov/pubmed/25205811.

(22) Bhattacharya S, Brincat SL, Lundqvist M, Miller EK. Traveling waves in the prefrontal cortex during working memory. PLoS Comput Biol. 2022;18(1):e1009827.

(23) Rubino D, Robbins KA, Hatsopoulos NG. Propagating waves mediate information transfer in the motor cortex. Nature Neuroscience. 2006;9(12):1549-57. Available from: https://doi.org/10.1038/nn1802.

(24) Davis ZW, Benigno GB, Fletterman C, Desbordes T, Steward C, Sejnowski TJ, et al. Spontaneous travelling waves naturally emerge from horizontal fiber time delays and travel through locally asynchronous-irregular states. Nature Communications. 2021;12(1):6057.

(25) Vogels TP, Abbott LF. Signal propagation and logic gating in networks of integrate-and-fire neurons. Journal of Neuroscience. 2005;25(46):10786-95.

(26) Kumar A, Schrader S, Aertsen A, Rotter S. The high-conductance state of cortical networks. Neural Computation. 2008;20(1):1-43.

(27) Destexhe A, Contreras D. Neuronal computations with stochastic network states. Science. 2006;314(5796):85-90.
